# Changes in the Glittre-ADL test in patients with non-small cell lung cancer: Pre- and postoperative analysis after home-based rehabilitation: A preliminary study

**DOI:** 10.1016/j.heliyon.2024.e40646

**Published:** 2024-11-22

**Authors:** Isabelle da Nobrega Ferreira, Joao Pedro Lima de Almeida, Mel Portugal Cabral Santos, Beatriz Martins Gomes Cruz, Thiago Thomaz Mafort, Agnaldo José Lopes

**Affiliations:** a Post-Graduation Programme in Medical Sciences, School of Medical Sciences, State University of Rio de Janeiro (UERJ), Rio de Janeiro, Brazil; b Faculty of Physiotherapy, Augusto Motta University Centre (UNISUAM), Rio de Janeiro, Brazil; c Rehabilitation Sciences Post-Graduation Programme, Augusto Motta University Center (UNISUAM), Rio de Janeiro, Brazil; d Local Development Post-Graduation Programme, Augusto Motta University Center (UNISUAM), Rio de Janeiro, Brazil

**Keywords:** Lung cancer, Rehabilitation, Functional capacity, Muscle strength, Lung function

## Abstract

**Background and objective:**

Postoperative pulmonary rehabilitation in non-small cell lung cancer (NSCLC) patients following thoracic surgery can be an important strategy for restoring functional exercise capacity. This study aimed to evaluate the changes in the Glittre-ADL test (TGlittre) in patients with NSCLC undergoing thoracic surgery and early home-based pulmonary rehabilitation and, subsequently, to assess the associations of the test results with muscle strength and quality of life (QoL).

**Methods:**

This observational study evaluated 18 patients with NSCLC before and after home-based pulmonary rehabilitation. Before thoracic surgery and 3 months after pulmonary rehabilitation, the participants underwent the following assessments: St. George's Respiratory Questionnaire (SGRQ), spirometry, measurement of the diffusion capacity of the lung for carbon monoxide (DLCO), respiratory muscle strength, handgrip strength (HGS), quadriceps muscle strength, and TGlittre.

**Results:**

When comparing the preoperative and postpulmonary rehabilitation periods, there was a significant increase in HGS [21.6 (19–29) vs. 28.5 (26–33) kgf, p = 0.011] with preservation of TGlittre time [3.6 (3.2–4) vs. 3.6 (3–4.1) min, *p* = 0.87]. In addition, there was a significant decrease in lung function and SGRQ scores. Preoperative TGlittre time significantly correlated with maximum inspiratory pressure (MIP, *r*_*s*_ = −0.491, *p* = 0.038) and DLCO (*r*_*s*_ = −0.621, *p* = 0.006). TGlittre time measured in postpulmonary rehabilitation was significantly correlated with HGS (*r*_*s*_ = −0.664, *p* = 0.002) and the MIP (*r*_*s*_ = −0.478, *p* = 0.045).

**Conclusion:**

In patients with NSCLC undergoing thoracic surgery and pulmonary rehabilitation, there was an increase in muscle strength and preservation of functional exercise capacity, although there was a decrease in lung function and QoL. There were interrelations of TGlittre time with MIP and pulmonary diffusion in the preoperative period and of TGlittre time with HGS and MIP following the home-based pulmonary rehabilitation. Although the results are promising, additional studies with a larger number of patients and a control group are needed to further support these findings.

## 1 Introduction

According to current estimates of global mortality, noncommunicable diseases are the leading cause of death in people aged 30–70 years, with cardiovascular disease being the leading cause, followed by cancer. These noncommunicable diseases have been identified as the leading cause of death in 127 countries, 57 of which have identified cancer as the leading cause of death, with lung cancer being the most common [1]. Despite this increase in prevalence, improvements in early preoperative staging, advances in surgical techniques, and more effective adjuvant treatment have increased survival [2]. With current surgical techniques, postoperative pulmonary complications occur in 20–30 % of patients and are considered the major cause of prolonged hospital stays, increased healthcare costs and poor quality of life (QoL) [3]. In addition, patients with non-small cell lung cancer (NSCLC) who undergo thoracic surgery experience physical deconditioning, muscle weakness, general fatigue, cachexia, and anxiety, which result in disability and decreased QoL [3].

In patients with NSCLC undergoing thoracic surgery, the supine position and general anesthesia induce cranial deviation of the diaphragm and reduce ventilatory efficiency, and the cough reflex is abolished, leading to atelectasis in dependent lung regions [4]. There is increasing evidence that pulmonary rehabilitation can benefit patients following thoracic surgery [5]. Comprehensive pulmonary rehabilitation has become a part of integrated care for these patients, where poor exercise tolerance is associated with poor postoperative outcomes and reduced survival [6]. Therefore, physical fitness should be assessed using specific measurement tools to guide exercise prescription and to evaluate the effectiveness of pulmonary rehabilitation programs. Pulmonary rehabilitation and exercise programs have been shown to reduce symptoms and increase exercise tolerance [3]. In addition to preoperative pulmonary rehabilitation, early mobilization and respiratory interventions are now routinely provided as part of the postoperative clinical plan; however, unfortunately, most patients do not receive ongoing pulmonary rehabilitation beyond the immediate postoperative period [7]. Despite recommendations for intervention as early as possible [7], studies using postsurgical rehabilitation have initiated exercise interventions after 3 weeks [[8], [9], [10], [11]]. A meta-analysis on the effects of postsurgical rehabilitation in patients with NSCLC showed that the patients underwent interventions in a hospital setting after discharge, and traveling made it difficult to adhere [2]. Brocki et al. [8] and Arbane et al. [11] evaluated the effects of postsurgical rehabilitation using QoL as the primary outcome, with little attention given to objective measures. During the late postoperative period after discharge, most patients are currently left to their own devices for exercise progression and physical activity [2]. It remains unclear which tools should be used for pre- and postoperative monitoring and evaluation, including assessment of the effects of pulmonary rehabilitation.

Pulmonary function tests (PFTs) are among the most widely used tools to assess postoperative outcomes and pulmonary complications in patients with NSCLC. The most commonly used functional parameter in clinical practice is the forced expiratory volume in 1 s (FEV_1_), which alone is a poor reflection of pulmonary pathophysiology, especially in patients with airflow obstruction [4]. In fact, there may be minimal loss or even improvement in lung function after thoracic surgery, especially in patients with an obstructive pattern undergoing lung resection, which casts doubt on the value of PFTs in the pre- and postoperative evaluation in these patients [12]. A possible explanation for these findings is the ‘volume reduction effect,’ where resection of the most affected parenchyma can lead to an improvement in the elastic recoil of the lung, a reduction in airflow resistance and an improvement in pulmonary mechanics and air‒blood coupling [13]. In addition to the fact that PFT values do not correlate with other functional parameters and QoL, they may be inadequate in the follow-up of patients with NSCLC following thoracic surgery and pulmonary rehabilitation, thus elevating the importance of an additional outcome measure [4].

In addition to PFTs, exercise testing is increasingly used to assess aerobic reserve in thoracic surgery candidates and to predict the occurrence of pulmonary complications [13]. Cardiopulmonary exercise testing (CPET) is indicated for functional assessment and preoperative risk assessment, as it provides several predictive measures of pulmonary complications in addition to postoperative assessment. However, CPET requires expensive equipment and experienced personnel and is rarely available [6]. When the CPET is not available or to complement the assessment of functional exercise capacity, other functional tests can be used to assess activities of daily living (ADLs). In 2006, the Glittre-ADL test (TGlittre) was developed to address the need for a broader and more representative objective assessment of physical function using ADL-like tasks [14]. The TGlittre requires mobility of both the lower and upper limbs as well as the pelvic and shoulder girdles [15]. Thus, the TGlittre is a field test that more reliably represents ADLs because it includes tasks such as walking, sitting, standing, going up and down stairs, reaching for objects, holding them with the hands, transferring objects of everyday weight, and squatting [16]. In preoperative patients with any type of lung disease, poorer performance on the TGlittre is associated with worsening gas exchange and body imbalance and may also serve as a prognosis for pulmonary complications, particularly with regard to chest tube duration [17].

The pulmonary rehabilitation needs in individuals undergoing thoracic surgery are changing, particularly as surgical management is more commonly offered to patients who are at risk of developing functional limitations. Pulmonary rehabilitation improves functional exercise capacity and reduces symptoms in patients with NSCLC, although the long-term effects on functional exercise capacity are unknown [2,18]. Although PFTs continue to be used primarily in the postoperative patient follow-up after thoracic surgery and pulmonary rehabilitation, exercise testing should also be performed to capture effects on functional exercise capacity that goes well beyond the isolated analysis of lung function [12]. The ideal test used for this purpose should be simple, inexpensive and widely available, which are characteristics of the TGlittre [14]. Indeed, the TGlittre has potential for clinical use in the patient follow-up after thoracic surgery, as it simulates postoperative stress and detects potential deficits in the lung, heart, and skeletal muscle [17]. We hypothesized that TGlittre is an important tool in the preoperative assessment of patients with NSCLC and that the TGlittre time decreases after postoperative home pulmonary rehabilitation. Therefore, this study aimed to evaluate the changes in TGlittre in patients with NSCLC undergoing thoracic surgery and early-initiated home-based pulmonary rehabilitation and, subsequently, to assess the associations of their results with muscle strength and QoL.

## 2 Materials

### 2.1 Subjects

Between August 2023 and July 2024, an observational study was conducted in patients with NSCLC aged ≥18 years who underwent thoracic surgery at the Pedro Ernesto University Hospital of the State University of Rio de Janeiro, Rio de Janeiro, Brazil. The following inclusion criteria were adopted: patients diagnosed with stage I or II NSCLC, i.e., patients without evidence of mediastinal disease or local organ invasion [19]; patients who underwent lung resection surgery, such as pneumonectomy, bilobectomy, lobectomy, segmentectomy, or wedge resection; or patients who underwent open thoracotomy or robotic-assisted thoracoscopic surgery. The exclusion criteria were as follows: severe dyspnea; intense pain; history of unstable angina; myocardial infarction less than 3 months before surgery; decompensated heart failure; use of an enteral tube; inability to walk; inability to perform PFTs and/or TGlittre; presence of neurological or orthopedic diseases; neoadjuvant therapy; and bronchopleural fistula or chylothorax during the postoperative period.

All participants provided written informed consent. The protocol was approved by the Research Ethics Committee of the Pedro Ernesto University Hospital of the State University of Rio de Janeiro, Rio de Janeiro, Brazil, under number CAAE-67676823.4.0000.5259. The trial has been registered on ClinicalTrials.gov under the number NCT05863013.

### 2.2 Pulmonary rehabilitation

Once admitted to the hospital, participants received intensive perioperative pulmonary rehabilitation. Each session lasted 2 h and consisted of therapeutic education, aerobic exercise, lower limb, upper limb and abdominal resistance training, and respiratory muscle training. After thoracic surgery, the participants underwent home-based pulmonary rehabilitation, which started within 2 weeks after lung resection and continued three times a week for 12 weeks. The participants received exercise prescription through a booklet with illustrations and descriptions of all the exercises, in addition to an exemplary video (.mp4 format) available on mobile phones to improve accessibility. On the day of instruction, the participants performed the exercises under the guidance of the physiotherapist.

Each home rehabilitation session consisted of muscle strengthening (including respiratory muscles), cardiorespiratory resistance, and flexibility exercises for 1 h. The session began with 5 min of warm-up exercises in which the patient was instructed to stretch their muscles (sternocleidomastoid, pectoral, broad dorsal, adductor magnus, biceps femoris, lateral deltoid, trapezius, external oblique, posterior thigh, hamstrings, calves, quadriceps, and lumbar regions) as tolerated, with each stretch held for 20–30 s. This was followed by 10 min of strengthening and resistance exercises for the large muscle groups of the upper and lower limbs (such as flexors, extensors, adductors, abductors, and rotators), with lateral and frontal elevation and flexion and extension exercises associated with functional diagonal movements, including exercises in open and closed kinetic chains. This was followed by 10 min of squatting, dorsiflexion and plantiflexion exercises using objects such as light weights, chairs, stairs and the walls; during the last 3 min of this period, the subjects were instructed to perform hand exercises consisting of flexion and extension of the fingers; abduction and adduction of the fingers in opposition to the thumb; flexion, extension, and ulnar and radial deviation of the wrist; and protonation and supination of the forearm. This was followed by 10 min of postural control training with proprioceptive exercises on the floor, followed by 20 min of cardiorespiratory training in functional circuits, considering the muscle groups commonly used in the patient's daily life. Finally, 5 min of relaxation and stretching with calisthenic exercises for the upper and lower limbs were performed [20,21].

The patients were contacted weekly by a physiotherapist to monitor the progress of the exercises. Home exercise intensity was monitored using Borg's Perceived Exertion Scale <4 and peripheral oxygen saturation >92 % as criteria; if these indicators were out of the range, the patient was instructed to stop pulmonary rehabilitation and contact the physiotherapist to restart pulmonary rehabilitation after 30 min [22].

### 2.3 Measurements

Assessments were performed at hospital admission before thoracic surgery and preoperative rehabilitation and again 3 months postop.

Physical activity was assessed via the short version of the International Physical Activity Questionnaire (IPAQ) [23]. The IPAQ consists of eight open-ended questions that assess the time and frequency of walking and moderate- and vigorous-intensity activities in the past week. Based on the IPAQ, participants were categorized as sedentary, not regularly active, active, or very active [23].

QoL was assessed via the St. George's Respiratory Questionnaire (SGRQ), which was previously adapted and validated for the Brazilian population [24]. The SGRQ has three domains: symptom, activity, and impact of the disease on the patient's daily life. The responses are translated into scores that, once added, can infer the QoL in a given domain. Scores above 10 % reflect a change in QoL.

PFTs were performed on an HDpft 3000 device (nSpire Health, Inc., Longmont, CO, USA), which included spirometry, measurement of the diffusion capacity of the lung for carbon monoxide (DLCO), and measurement of respiratory muscle strength. Examination protocols followed previously established recommendations [25,26]. National equations were used to calculate the predicted values for each participant [[27], [28], [29]]. The obstructive pattern, restrictive pattern, and pulmonary diffusion impairment were defined and/or suggested by a reduced ratio of FEV_1_ to forced vital capacity (FEV_1_/FVC), reduced FVC in the absence of airflow obstruction, and reduced DLCO, respectively [30].

Handgrip strength (HGS) was measured in kilograms using a digital handheld dynamometer (SH5001, Saehan Corporation, Korea). HGS was assessed with participants seated in an armless chair with the elbow flexed at 90°, forearms in the neutral position, and wrist extension between 0° and 30° [31]. Maximal strength was measured after a sustained 3-s contraction in the dominant hand; the highest value of three trials at 1-min intervals was used for analysis. The cutoff points used were 27 kgf for men and 16 kgf for women, as previously proposed [32].

Quadriceps muscle strength (QMS) was assessed via a traction dynamometer with a sensor capacity of 200 kg (E-lastic 5.0, E-sporte SE, Brazil). The knee during the test was at 90°, starting with the knee in flexion. Maximum strength was assessed after a sustained 5-s contraction of the dominant leg, and the highest value of 3 trials with 1-min intervals was included for analysis [33]. The cutoff points used were 25.3 kgf for men and 14.8 kgf for women [34].

TGlittre was performed as described by Skumlien et al. [14]. It is a circuit of functional activities in a 10-m corridor to be completed by the participant in the shortest possible time, carrying a backpack weighing 2.5 kg for women and 5 kg for men. In this circuit, the participant walks from a sitting position on a flat path with a box in the middle, with two steps to go up and two steps to go down. After completing the rest of the path, the participant encounters a shelf containing three objects, each weighing 1 kg, placed on the highest shelf (shoulder height). They must then move them one at a time to the lowest shelf and then to the floor. The objects must then be placed back on the lowest shelf and then on the highest shelf. The participant then returns and completes the circuit in reverse, immediately starting a new round and completing the same circuit. To complete the TGlittre, the participant must complete 5 laps in the shortest possible time. At the beginning and at the end of the test, the Borg scale was used to assess the level of perceived exertion. The protocol was performed twice with an interval of 30 min, and the shortest TGlittre was used for analysis and compared with the predicted Brazilian values [35].

### 2.4 Statistical analysis

Statistical analysis was performed with Statistical Package for Social Sciences (SPSS) version 26 (IBM Corp., Armonk, NY, USA). The normality of the data was assessed using the Shapiro‒Wilk test and graphical analysis of histograms. Variation between the two time points (preoperative period and after home-based rehabilitation) was assessed using Student's *t*-test for paired samples or the Wilcoxon signed-rank test for numerical data and the McNemar test for categorical data.

Because the participants underwent two approaches with opposite effects on physical function (thoracic surgery vs. pulmonary rehabilitation), we evaluated correlations and associations in two different periods: preoperative and following home-based rehabilitation. Thus, the TGlitree time (% predicted) measured in the preoperative period was correlated with variables obtained in the same period and with chest tube duration and length of hospital stay using Spearman's correlation coefficient (numerical variables) or the Wilcoxon‒Mann‒Whitney test (categorical variables). TGlittre time (% predicted) measured following the home-based rehabilitation period was correlated with variables obtained in the same period and with chest tube duration and length of hospital stay via Spearman's correlation coefficient (numerical variables) or the Wilcoxon‒Mann‒Whitney test (categorical variables). Multivariate analysis by multiple linear regression was used to identify the independent variables that explained the variability in the logarithm of TGlittre time (% predicted) in the preoperative period and in the post-rehabilitation period. The variable selection process was forward stepwise at the 5 % level, which selects the smallest subset of independent variables that best explains the dependent variable (TGlittre time, % predicted). This analysis was applied to the data with natural logarithmic transformation to adapt the distribution to a parametric approach. The criterion used to determine significance was the 5 % level.

To contextualize the interpretation of the results, a *post hoc* power analysis was performed via G∗Power statistical program version 3.1.9.6 (Heinrich Heine University, Düsseldorf, Germany) based on the variations in the parameters between the two time points (preoperative and post-rehabilitation period).

## 3 Results

Of the 20 NSCLC patients aged ≥18 years who underwent thoracic surgery, two were excluded because they were unable to complete the preoperative TGlittre. Thus, 18 participants completed the study with pre- and postoperative assessments, with a median postoperative time of 3 (3–4) months. Among these, 11 (61.1 %) were women, with a median age of 62 (51–70) years. Fourteen (77.8 %) patients had a history of smoking, with a median smoking history of 12 (2–47) years. Six (33.3 %) participants were sedentary, 8 (44.4 %) were not regularly active, and 4 (22.2 %) were active. Fifteen (83.3 %) participants underwent open thoracotomy, and the most common resection size was wedge/segmentectomy, which was performed in 10 (55.6 %) participants. The most common histologic types were adenocarcinoma and squamous cell carcinoma in 11 (61.1 %) and 4 (22.2 %) participants, respectively. The sociodemographic characteristics, comorbidities, surgical procedure data, chest tube duration, and length of hospital stay of the participants are shown in Table 1.

**Table 1:** Sociodemographic parameters, comorbidities, and surgical procedure data.

Variable	Values
**Sociodemographic**	
Male/female	7/11
Age (years)	62 (51–70)
Weight (kg)	67.7 ± 13.2
Height (m)	1.63 ± 0.08
BMI (kg/m^2^)	25.5 ± 3.9
**Comorbidities**	
Hypertension	8 (44.4 %)
Diabetes	6 (33.3 %)
**Surgical approach**	
Open thoracotomy	15 (83.3 %)
Robotic-assisted thoracoscopic surgery	3 (16.7 %)
**Resection size**	
Wedge/segmentectomy	10 (55.6 %)
Lobectomy/bilobectomy	6 (33.3 %)
Pneumonectomy	2 (11.1 %)
**NSCLC histology**	
Adenocarcinoma	11 (61.1 %)
Squamous	4 (22.2 %)
Large cell	1 (5.56 %)
Sarcomatoid carcinoma	1 (5.56 %)
NSCLC not otherwise specified	1 (5.56 %)
**Chest tube duration**	4 (3–6)
**Length of hospital stay**	24 (19–30)

Values are the median (interquartile range), mean ± SD, or number (%). BMI: body mass index; NSCLC: non-small-cell lung cancer.

At the preoperative evaluation of lung function, normal, restrictive and obstructive patterns were observed in 10 (55.6 %), 4 (22.2 %) and 4 (22.2 %) participants, respectively, whereas 5 (27.8 %) of them had reduced DLCO. At the post-pulmonary rehabilitation evaluation of lung function, normal patterns, restrictive patterns, and obstructive patterns were observed in 6 (33.3 %), 8 (44.4 %), and 4 (22.2 %) participants, respectively, whereas 6 (33.3 %) of them had reduced DLCO. When comparing the preoperative and post-pulmonary rehabilitation evaluations, there was a significant reduction in the FVC (94.2 ± 16.2 vs. 79.2 ± 14.6 % predicted, *p* = 0.0001), FEV_1_ (87.4 ± 18.4 vs. 77.7 ± 17 % predicted, *p* = 0.003) and peak expiratory flow (PEF, 87.2 ± 26.5 vs. 73.4 ± 20.4 % predicted, *p* = 0.037). In terms of QoL, the post-rehabilitation assessment revealed worsening scores in the ‘symptom’ [5.8 (0–13) vs. 17.2 (10–30), *p* = 0.017] and ‘activity’ [11.1 (0–24) vs. 20.3 (5–49), *p* = 0.004] domains compared with the preoperative assessment, although no changes were observed in the ‘impact’ domain. Comparisons of lung function and QoL between the preoperative and postrehabilitation periods are shown in Table 2.

**Table 2:** Comparisons of lung function and quality of life between the preoperative and post-pulmonary rehabilitation periods.

	Preoperative period	Home-based post-pulmonary rehabilitation period	*p*-value
**Pulmonary function**			
FVC (% predicted)	94.2 ± 16.2	79.2 ± 14.6	**0.0001**
FEV_1_ (% predicted)	87.4 ± 18.4	77.7 ± 17	**0.003**
FEV_1_/FVC (%)	78.8 ± 10.6	80.2 ± 7.5	0.53
PEF (% predicted)	87.2 ± 26.5	73.4 ± 20.4	**0.037**
DLCO (% predicted)	96 ± 25	93.9 ± 25.4	0.66
**SGRQ**			
Score-symptom	5.8 (0–13)	17.2 (10–30)	**0.017**
Score-activity	11.1 (0–24)	20.3 (5–49)	**0.004**
Score-impact	10 (6.3–21)	11.2 (4.5–29)	0.68
Score-total	12.1 (4.5–18)	17.3 (6.1–32)	**0.015**

Values are mean ± SD or median (interquartile range). FVC: forced vital capacity; FEV_1_: forced expiratory volume in 1 s; PEF: peak expiratory flow; DLCO: diffusion capacity of lung for carbon monoxide; SGRQ: St George's Respiratory Questionnaire.

In the preoperative muscle strength assessment, the maximum inspiratory pressure (MIP) and maximum expiratory pressure (MEP) were reduced in 10 (55.6 %) and 14 (77.8 %) participants, respectively. In addition, the HGS and QMS were reduced in 4 (22.2 %) and 5 (27.8 %) participants, respectively. In the post-rehabilitation assessment of muscle strength, the MIP and MEP decreased in 6 (33.3 %) and 10 (55.6 %) participants, respectively. Further, the HGS and QMS were lower in 1 (5.56 %) and 1 (5.56 %) participant, respectively. When the preoperative and post-rehabilitation evaluations were compared, only HGS significantly increased [21.6 (19–29) vs. 28.5 (26–33) kgf, *p* = 0.011]. Regarding TGlittre time, 11 (61.1 %) participants had values >120 % predicted at the preoperative evaluation, whereas 8 (44.4 %) participants had values >120 % predicted at the post-rehabilitation evaluation. When comparing the preoperative and post-rehabilitation evaluations, there was no change in TGlittre time, either in terms of absolute values or values in % predicted. Comparisons of muscle strength and functional exercise capacity between the pre- and post-rehabilitation evaluations are shown in Table 3.

**Table 3:** Comparisons of muscle strength and functional exercise capacity between the preoperative and post-pulmonary rehabilitation periods.

	Preoperative period	Home-based post-pulmonary rehabilitation period	*p*-value
**Muscle strength**			
MIP (% predicted)	64.7 ± 21.2	70.5 ± 20.5	0.26
MEP (% predicted)	52.2 ± 16.6	54.7 ± 16.2	0.62
HGS (kgf)	21.6 (19–29)	28.5 (26–33)	**0.011**
QMS (kgf)	31.1 ± 11.1	33.5 ± 10	0.19
**Functional exercise capacity**			
TGlittre time observed (min)	3.6 (3.2–4)	3.6 (3–4.1)	0.87
TGlittre time (% predicted)	124 (108–134)	119 (111–133)	0.91

Values are mean ± SD or median (interquartile range). PR: pulmonary rehabilitation; MIP: maximum inspiratory pressure; MEP: maximum expiratory pressure; HGS: handgrip strength; QMS: quadriceps strength; TGlittre: Glittre-ADL test.

**Fig. 1:**
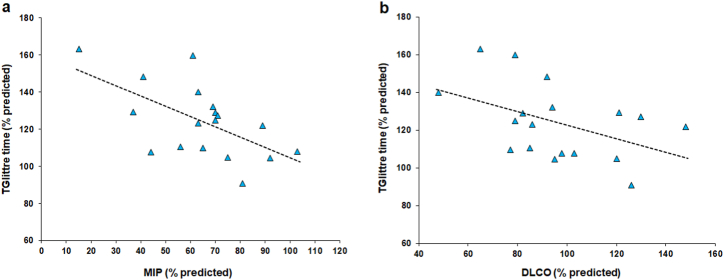
Relationships of Glittre-ADL test (TGlittre) time in the preoperative period with maximum inspiratory pressure (MIP) (a) and diffusion capacity of lung for carbon monoxide (DLCO) (b) in patients with non-small cell lung cancer.

Preoperative TGlittre time significantly correlated with MIP (*r*_*s*_ = −0.491, *p* = 0.038) and DLCO (*r*_*s*_ = −0.621, *p* = 0.006). There were no significant associations between preoperative TGlittre time and the following variables: sex, comorbidities, surgical approach, type of resection, NSCLC histology, chest tube duration, or length of hospital stay (Table 4 and Fig. 1). In the multiple linear regression for the log of TGlitree time measured in the preoperative period (% predicted), the MIP was the only independently predictive variable, explaining 34 % of its variability (Table 5).

**Table 4:** Spearman's correlation coefficients for the Glittre-ADL test, sociodemographic parameters, lung function, muscle strength, quality of life, and surgical procedure data.

	TGlittre time			
	Preoperative period		Home-based post-pulmonary rehabilitation period	
	r_s_	*p-*value	r_s_	*p-*value
Age	0.021	0.93	0.104	0.68
Weight	−0.121	0.63	−0.360	0.14
Height	−0.082	0.75	−0.052	0.84
BMI	−0.009	0.97	−0.359	0.14
Smoking load	0.257	0.30	0.161	0.52
FVC	−0.106	0.67	−0.239	0.34
FEV_1_	−0.297	0.23	−0.090	0.72
FEV_1_/FVC	−0.377	0.12	−0.094	0.71
PEF	−0.174	0.49	−0.366	0.14
DLCO	**−0.491**	**0.038**	−0.127	0.62
MIP	**−0.621**	**0.006**	**−0.478**	**0.045**
MEP	−0.308	0.21	−0.267	0.28
HGS	−0.237	0.34	**−0.664**	**0.002**
QMS	−0.316	0.20	−0.381	0.12
Score-symptom	0.075	0.77	0.183	0.47
Score-activity	0.203	0.42	0.137	0.59
Score-impact	0.033	0.90	0.266	0.29
Score-total	0.149	0.56	0.236	0.29
Chest tube duration	0.049	0.85	0.316	0.20
Length of hospital stay	0.086	0.73	0.312	0.21

TGlittre: Glittre-ADL test; BMI: body mass index; FVC: forced vital capacity; FEV_1_: forced expiratory volume in 1 s; PEF: peak expiratory flow; DLCO: diffusion capacity of lung for carbon monoxide; MIP: maximum inspiratory pressure; MEP: maximum expiratory pressure; HGS: handgrip strength; QMS: quadriceps strength; SGRQ: St George's Respiratory Questionnaire.

**Table 5:** Independent linear model for the Glittre-ADL test using sociodemographic parameters, lung function, muscle strength, quality of life, and surgical procedure data.

	B	SEB	*P* value	R	Adjusted R^2^
**TGlittre time in the preoperative period**					
Constant	5.008	0.102	<0.0001		
MIP	−0.004	0.001	0.011	0.58	0.34
**TGlittre time in the home-based post-PR period**					
Constant	5.506	0.209	<0.0001		
HGS	−0.024	0.007	0.004	0.64	0.41

B: regression coefficient; SEB: standard error of the regression coefficient; R: cumulative correlation coefficient; R^2^: cumulative adjusted coefficient of determination; TGlittre: Glittre-ADL test; MIP: maximum inspiratory pressure; HGS: handgrip strength.

**Fig. 2:**
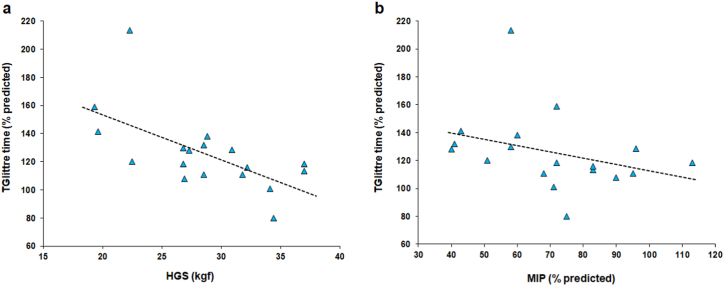
Relationships of Glittre-ADL test (TGlittre) time in the home-based post-pulmonary rehabilitation period with handgrip strength (HGS) (a) and maximum inspiratory pressure (MIP) (b) in patients with non-small cell lung cancer.

In the post-rehabilitation period, the TGlittre time measured significantly correlated with HGS (*r*_*s*_ = −0.664, *p* = 0.002) and MIP (*r*_*s*_ = −0.478, *p* = 0.045). There were no significant associations between TGlitree time and the following variables: sex, comorbidities, surgical approach, type of resection, NSCLC histology, chest tube duration, or length of hospital stay (Table 4 and Fig. 2). In the multiple linear regression for log TGlittre time measured at the post-rehabilitation evaluation (% predicted), HGS was the only independent predictive variable, explaining 41 % of its variability (Table 5).

Based on an *a priori* type I error of α = 0.05 (two-tailed), the power analysis revealed significant effects on the variations in the parameters between the two time points (preoperative period and post-rehabilitation period). The power varied between 62 % and 93 %, indicating that significant results were achieved even with a small sample [36].

## 4 Discussion

Lung resection surgery results in a reduction in physical fitness that does not fully recover over time, which can potentially have a major impact on lung function and ADLs in patients. In addition to the need to understand the impact on postoperative function, there is an increasing need to implement postoperative rehabilitation strategies in patients with NSCLC to reduce the negative impact. The primary findings of this study were that although there was a decrease in lung function and QoL following home-based postpulmonary rehabilitation, the TGlittre time remained close to the preoperative values. When comparing the preoperative and post-rehabilitation evaluations, there were gains in respiratory and peripheral muscle strength, especially HGS. Furthermore, TGlittre time in the preoperative period was associated with MIP and DLCO, whereas TGlittre time in the post-rehabilitation period was associated with HGS and MIP. To our knowledge, this is the first study to demonstrate the long-term impact of pulmonary rehabilitation in patients with NSCLC using TGlittre as an assessment tool for functional exercise capacity.

Studies on exercise training for patients with NSCLC have focused on facility-based exercise programs, although patients prefer to exercise at home [37]. It is important for all patients to adhere to the pulmonary rehabilitation protocol, which can be improved, at least in part, by addressing issues such as availability and transportation related to facility-based rehabilitation. We observed a decrease in the FVC, FEV_1_, and PEF values following home-based pulmonary rehabilitation, which is expected since these patients lost a significant part of the parenchyma, and rehabilitation has not been shown to improve lung function [38]. However, the decline in lung function appears to be attenuated with rehabilitation when these patients are compared with matched controls [39]. In evaluating the effect of pulmonary rehabilitation in patients with NSCLC in the perioperative period of thoracoscopic lobectomy, Zhou and Sun [39] reported that the PEF in the treatment group was greater than that of controls at 21 days after surgery, and the FEV_1_ at 28 days after surgery was greater than that of controls. Since lung function is closely related to structural changes in the lung, the impact of pulmonary rehabilitation on lung mechanics is small [40]. Therefore, surgical techniques that can reduce lung damage should be used whenever possible, such as robotic-assisted thoracoscopic surgery [41,42], which was performed in less than 20 % of our participants. Notably, we observed that surgical resection had a detrimental effect on the QoL of patients with NSCLC, which was not improved by rehabilitation. In fact, there is a high prevalence of pain, fatigue, and dyspnea after surgery, and most patients report a deterioration of their physical and emotional health postoperatively, with a negative impact on QoL [43]. Thus, psychosocial and behavioral interventions should be added to pulmonary rehabilitation by incorporating educational training, cognitive behavioral therapy, and support groups focused on stress management and progressive muscle relaxation [5].

Impaired physical fitness and muscle loss combined with high symptom burden are likely associated with avoidance of physical activity in the postoperative period following thoracic surgery. To this end, we aimed to fill a gap in the literature regarding an in-depth investigation on the effects of long-term postoperative pulmonary rehabilitation on respiratory and peripheral muscle strength. Although there were increases in respiratory and peripheral muscle strength between the preoperative and post-rehabilitation evaluations, a significant increase was observed only in HGS. Since HGS is a clinical marker of poor physical performance and all-cause mortality [44], reconditioning programs should focus on restoring muscle function as early as possible. In addition, HGS is a simple, noninvasive and inexpensive measurement that can be used as a biomarker in the monitoring of several types of cancer, including NSCLC [44]. Tough et al. [45] recently evaluated the postoperative outcomes in patients who underwent surgical resection of NSCLC in the last 3 months. The authors reported that dynamic balance and gait were impaired compared to age-matched healthy controls, including a smaller proportion performing moderate and vigorous physical activity. This may be a consequence of the impaired muscle strength that these patients present in the postoperative period, emphasizing the need to promote early ambulation and the use of rehabilitation strategies aimed at restoring muscle strength [7].

The literature highlights that, after lung resection, patients have a more complete recovery of functional exercise capacity than of pulmonary mechanics and gas exchange capacity because of several compensatory mechanisms related to the cardiovascular system and peripheral oxygen extraction capacity [9]. In fact, we observed no change in functional exercise capacity assessed by TGlittre when we compared the median values among the three months, indicating that home-based pulmonary rehabilitation prevented an expected decline in functional exercise capacity related to the negative effects of thoracic surgery. While more than 60 % of our patients had values >120 % predicted at the preoperative evaluation (indicating poor performance), only approximately 40 % of them had values >120 % predicted at the post-rehabilitation evaluation. Although postoperative exercise training can effectively improve functional exercise capacity, a longer training period is needed. A systematic review and meta-analysis revealed that the 6-min walk distance increased to 62.8 m after 12 weeks of training in patients with NSCLC [46]. Further, Tenconi et al. [47] reported that intensive pre- and postoperative pulmonary rehabilitation in patients with NSCLC undergoing thoracic surgery was able to restore functional exercise capacity at 6 months, as assessed by the 6-min walk distance.

In our study, the preoperative TGlittre time correlated significantly with the preoperative MIP and DLCO. Consistent with our results, da Nobrega Ferreira et al. [17] reported a significant correlation between TGlittre time and DLCO at the preoperative evaluation in patients eligible for thoracic surgery, in which one-third of them had NSCLC, However, these authors did not measure respiratory or peripheral muscle strength. The correlation between TGlittre time and DLCO is not surprising, as DLCO measures the transfer of inhaled gas between the alveoli and red blood cells within the pulmonary capillaries and therefore has a robust effect on functional exercise capacity [48]. Notably, our multiple linear regression model for TGlittre time in the preoperative period was explained only by the MIP, indicating the need for attention to the MIP in the rehabilitative care in these patients.

In our study, the TGlittre time post-rehabilitation significantly correlated with the HGS and MIP measured postoperatively. Huang et al. [37] also reported an association between functional exercise capacity and the MIP following postoperative rehabilitation in patients with NSCLC. After rehabilitation, functional exercise capacity improved significantly, as evidenced by increased maximal oxygen consumption and work rates. Breathing exercises, such as diaphragmatic breathing, aim to strengthen the diaphragm, and MIP measurements are sensitive and can detect even small changes in respiratory muscle strength [37]. Notably, our multiple linear regression model for TGlittre time following home-based pulmonary rehabilitation was explained only by HGS. Because TGlittre involves upper limb activity (which the 6-min walk test does not), this association between TGlittre and HGS may be partially explained by the inclusion of several hand muscle strengthening exercises in our home-based rehabilitation.

This study has several limitations. First, the sample size was small, which limits the statistical power and generalizability of the results, since we included patients with NSCLC who underwent thoracic surgery. Given the small sample size, the use of multiple statistical tests increases the risk of type I error [49]. Second, the lack of a control group makes it difficult to attribute changes to home-based pulmonary rehabilitation. It may be of great interest to compare the effects of postsurgical rehabilitation between a group that undergoes home-based pulmonary rehabilitation and a group that undergoes an outpatient pulmonary rehabilitation program. Third, our single-center design may have introduced selection bias, although our hospital is a reference for lung cancer in Brazil. A multicenter approach would improve the external validity. Fourth, we did not use TGlittre with dynamic ventilation measurements. More recently, the integration of the two assessment tools has been useful for elucidating pathophysiological mechanisms in other clinical conditions [50,51]. Finally, we did not use the CPET to assess functional exercise capacity because it requires expensive and specific equipment and specialized personnel, and does not represent the usual physical activity level of people with NSCLC [6]. Despite these limitations, our findings can serve as a foundation for future randomized controlled trials to evaluate postoperative home-based pulmonary rehabilitation and the use of TGlittre in pre- and postoperative management in more robust and multicentric samples of NSCLC patients. Future studies are needed to evaluate the long-term effects of home-based pulmonary rehabilitation (including comparisons with facility-based exercise programs) and to assess the potential of integrating other assessment tools into TGlittre (such as measurement of dynamic ventilation during testing) in this population.

## 5 Conclusion

In patients with NSCLC who underwent thoracic surgery and pulmonary rehabilitation, there was an increase in muscle strength and preserved functional exercise capacity, although there is a decrease in lung function and QoL. During the preoperative period, the lower the MIP and pulmonary diffusion were, the longer the TGlittre time was. During the postoperative at-home rehabilitation, the lower the HGS and MIP were, the longer the TGlittre time was. In these patients, the determinants of TGlittre time in the preoperative period and the postoperative at-home rehabilitation are possibly MIP and HGS, respectively. Although the results are promising, additional studies with a larger number of patients and a control group are needed to validate these findings.

## CRediT authorship contribution statement

**Isabelle da Nobrega Ferreira:** Writing – review & editing, Writing – original draft, Validation, Project administration, Formal analysis, Conceptualization. **Joao Pedro Lima de Almeida:** Writing – review & editing, Writing – original draft, Investigation, Data curation. **Mel Portugal Cabral Santos:** Writing – review & editing, Writing – original draft, Investigation, Data curation. **Beatriz Martins Gomes Cruz:** Writing – review & editing, Writing – original draft, Investigation, Data curation. **Thiago Thomaz Mafort:** Writing – review & editing, Writing – original draft, Validation, Supervision, Formal analysis, Conceptualization. **Agnaldo José Lopes:** Writing – review & editing, Writing – original draft, Validation, Supervision, Funding acquisition, Formal analysis, Conceptualization.

## Ethics approval and consent to participate

This study was approved by the Research Ethics Committee of the Pedro Ernesto University Hospital of the State University of Rio de Janeiro, Rio de Janeiro, Brazil, under protocol number CAAE-67676823.4.0000.5259. The trial was registered at ClinicalTrials.gov with reference number NCT05863013. All participants signed an informed consent form.

## Consent for publication

Not applicable.

## Data availability statement

Data will be made available on request.

## Author agreement/declaration

All authors reviewed and approved the final form of the submitted paper. The authors declare that this work is original and that all information, including all tables and figures, was generated by the authors, has not been published, and is not under consideration elsewhere.

## Funding

The authors wish to thank the Conselho Nacional de Desenvolvimento Científico e Tecnológico (10.13039/501100003593CNPq; Grant numbers #301967/2022-9 and #401633/2023-3), Brazil; the Fundação de Amparo à Pesquisa do Estado do Rio de Janeiro (10.13039/501100004586FAPERJ; Grant number #E−26/200.929/2022), Brazil, and the Coordenação de Aperfeiçoamento de Pessoal de Nível Superior (10.13039/501100002322CAPES, Finance Code 001), Brazil.

## Declaration of competing interest

The authors declare the following financial interests/personal relationships which may be considered as potential competing interests:No. If there are other authors, they declare that they have no known competing financial interests or personal relationships that could have appeared to influence the work reported in this paper.
